# Supplementary material for: Optimizing high-temperature energy storage in tungsten bronze-structured ceramics via high-entropy strategy and bandgap engineering
Source: Nat Commun. 2024 Jul 12;15:5869. doi: 10.1038/s41467-024-50252-w (PMC11245601; doi:10.1038/s41467-024-50252-w)
Supplement: Supplementary file 1 — Supplementary Information [file 41467_2024_50252_MOESM1_ESM.pdf]

Supplementary Information

Optimizing High-Temperature Energy Storage in Tungsten Bronze structured Ceramics via High-Entropy Strategy and Bandgap Engineering

Yangfei Gao [1#], Zizheng Song [2#], Haichao Hu [1], Junwen Mei [1], Ruirui Kang [1], Xiaopei Zhu [3], Bian Yang [3], Jinyou Shao [1, 4], Zibin Chen [2*], Fei Li [5], Shujun Zhang [6*], and Xiaojie Lou [1*]

[1] *Frontier Institute of Science and Technology, State Key Laboratory for Mechanical Behavior of Materials, and Xi'an Key Laboratory of Electric Devices and Materials Chemistry, Xi'an Jiaotong University, Xi'an 710049, China*

[2] *Department of Industrial and Systems Engineering, The Hong Kong Polytechnic University, Hong Kong, China*

[3] *School of Materials Science and Engineering, Xi'an University of Technology, Xi'an, Shaanxi 710048, China*

[4] *Micro-and Nano-Technology Research Center, State Key Laboratory for Manufacturing Systems Engineering, Xi'an Jiaotong University, Xi'an 710049, China*

[5] *Electronic Materials Research Laboratory (Key Lab of Education Ministry), State Key Laboratory for Mechanical Behavior of Materials and School of Electronic and Information Engineering, Xi'an Jiaotong University, Xi'an 710049, China.*

[6] *Institute for Superconducting and Electronic Materials, Faculty of Engineering and Information Sciences, University of Wollongong, Wollongong, NSW, Australia*

# These authors contributed equally: Yangfei Gao, Zizheng Song

**Corresponding author's email address: zi-bin.chen@polyu.edu.hk (Z.B. Chen), shujun@uow.edu.au (S.J. Zhang), xlou03@mail.xjtu.edu.cn (X.J. Lou)*

Formulas and theories

The modified Curie-Weiss equation[1]

$$\frac{1}{\varepsilon_r} - \frac{1}{\varepsilon_{rm}} = \frac{(T - T_m)^\gamma}{C}$$

where ε_r, ε_{rm}, C and γ mean the relative permittivity, maximum dielectric constant, Curie constant and diffusion coefficient, respectively.

The Vogel-Fulcher model can be expressed as [2]:

$$f = f_0 \exp\left(\frac{E_a}{k(T_m - T_f)}\right) \quad (1)$$

where f_0 is an attempt frequency, E_a the activation energy, k the Boltzmann constant, T_m the temperature at the maximum dielectric constant and T_f the freezing temperature.

Weibull distribution

The plot of Weibull distribution is described by Refs.[3]:

$$X_i = \ln(E_i) \quad (2)$$

$$Y_i = \ln(-\ln(1 - i/(\mathrm{n}+1))) \quad (3)$$

where n is the total number of samples of each content, β is the slop of each fitting straight line, E_i represents the E_b of each sample and i is the sequence number. Samples of each content are sorted by dielectric breakdown strength values:

$$E_1 \le E_2 \le \cdots \le E_i \le \cdots \le E_\mathrm{n}$$

Arrhenius formula.[4]:

$$\sigma = \sigma_0 exp(-E\mathrm{a}/kT) \quad (2)$$

where σ_0 is a pre-exponential factor, The activation energy of conduction *Ea* represents the ability of carriers to form and migrate, *k* is the Boltzmann constant, and *T* is the temperature in Kelvin.

Tauc plot.[5]

Tauc plot is mainly based on the formula proposed by Tauc, Davis and Mott et al.[5]: $(\alpha h\nu)^{1/n} = B(h\nu - E_{bg})$, where α is absorption coefficient, h is Planck-constant, *ν* is frequency, B is constant, Eg is the bandgap width of semiconductor, Exponential n is directly related to the type of semiconductor, direct bandgap n=1/2, indirect bandgap n=2. For calculating the direct bandgap, n = 1/2, $(Ah\nu)^2 = h\nu - E_{bg}$.

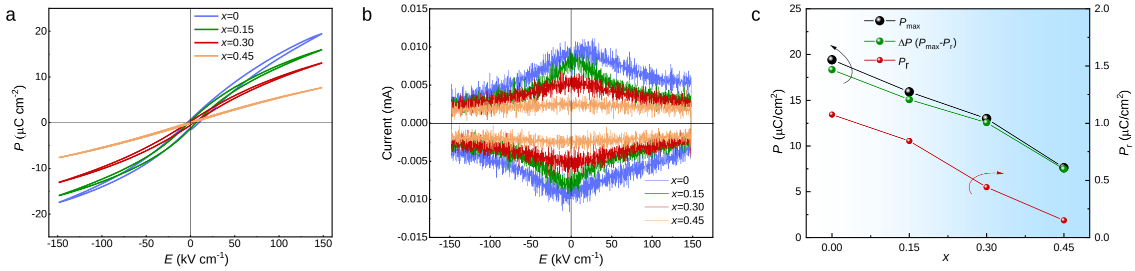

Figure S1. Ferroelectric properties of BSCNT*x*. (a)Bipolar P-E loops of BSCNT ceramics at 150 kV/cm. (b) The corresponding current-electric field (*I*-*E*) curves aligned with *P*-*E* loops. (c) P_{max}, P_r and ΔP of BSCNT ceramics at 150 kV/cm and 10Hz.

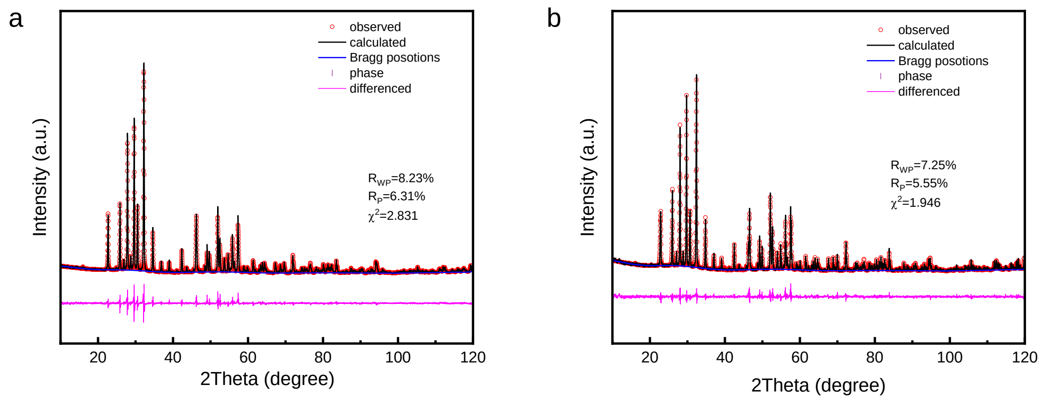

Figure S2. The Rietveld refinement results of XRD data. (a) BSCNT0, (b) BSCNT0.30.

Table S1. The cationic position and proportion of BSCNT0 ceramics obtained XRD refinement results

Name	Fractional coordinates			Occupancy
Ba2	0.172144	0.672144	0.503508	0.5152
Sr2	0.172738	0.672738	0.493875	0.3821
Sr1	0.000000	0.000000	0.489268	0.7082
Nb1	0.500000	0.000000	0.015835	0.9987
Nb2	0.074493	0.211493	0.001110	0.9971
O1	0.282125	0.782125	0.968522	1.0628
O2	0.138593	0.068118	0.958397	0.9683
O3	0.993724	0.341702	0.958619	1.0299
O4	0.500000	0.000000	0.478860	1.0045
O5	0.074595	0.205913	0.466276	1.0154

Table S2. The cationic position and proportion of BSCNT0.30 ceramics obtained XRD refinement results

Name	Fractional coordinates			Occupancy
Ba2	0.172491	0.672491	0.516609	0.5253
Sr2	0.172189	0.672190	0.510159	0.2802
Sr1	0.000000	0.000000	0.498482	0.1923
Ca1	0.000000	0.000000	0.490252	0.7053
Nb1	0.500000	0.000000	0.020352	0.8494
Ta1	0.500000	0.000000	0.020352	0.1533
Nb2	0.074552	0.211098	0.009753	0.8494
Ta2	0.074552	0.211098	0.009753	0.1533
O1	0.282076	0.782076	0.949705	1.0298
O2	0.140983	0.069492	0.948012	1.0371
O3	0.991005	0.338295	0.937655	0.9979
O4	0.500000	0.000000	0.525831	0.9737
O5	0.072395	0.205210	0.509134	1.0464

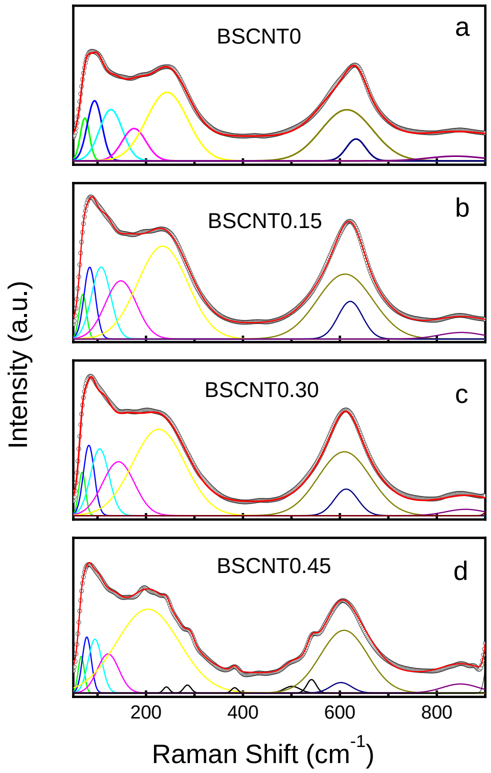

Figure S3. Raman spectra of BSCNT at room temperature and the fitting results using the Gauss-Lorentz function to analyze the peaks. (a)BSCNT0, (b)BSCNT0.15, (c)BSCNT0.30, (d)BSCNT0.45.

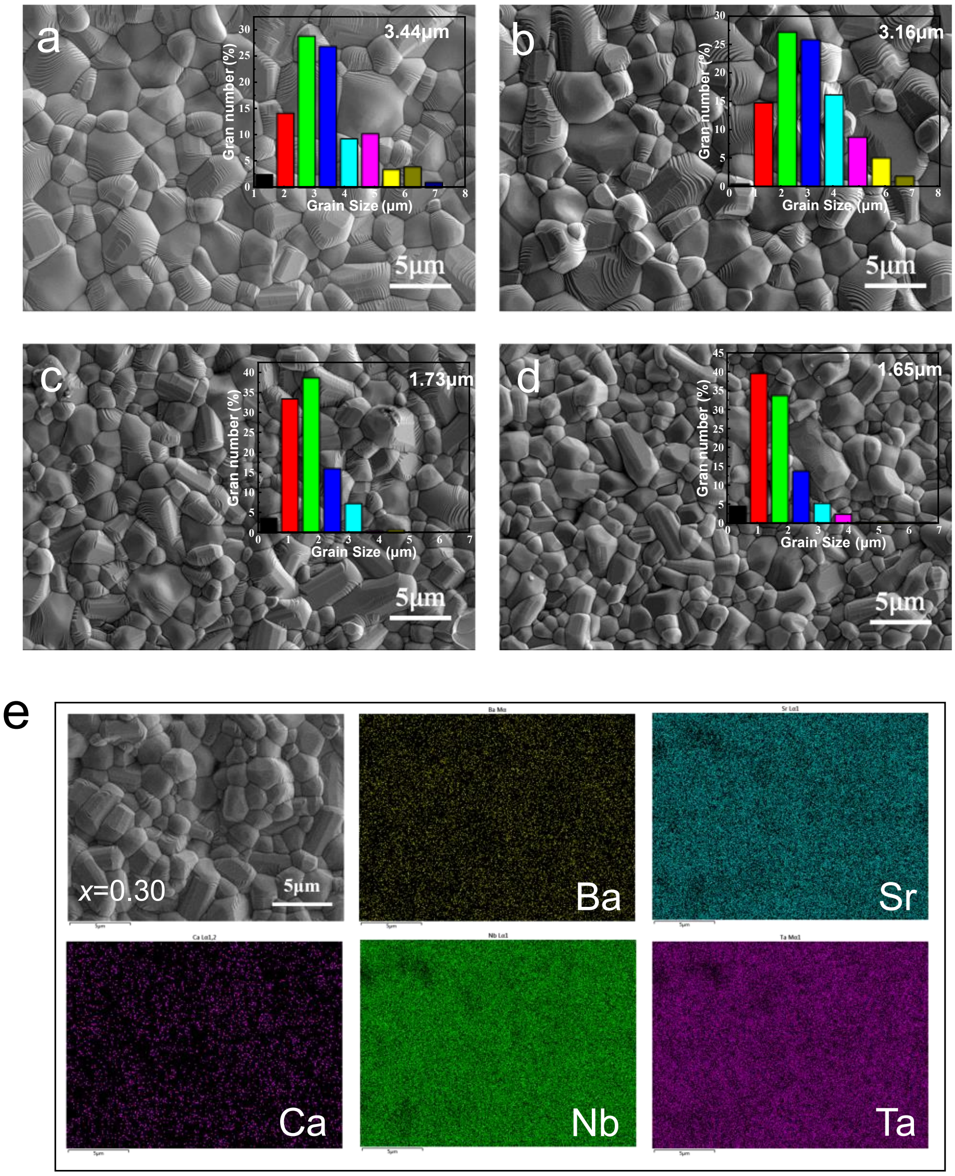

Figure S4. Surface morphology and grain size statistics of BSCNT*x* ceramics. (a)-(d) SEM images of the studied BSCNT samples, with their respective grain size distributions and average grain sizes (as shown in the inset), and (e) the elemental distribution map of BSCNT0.30.

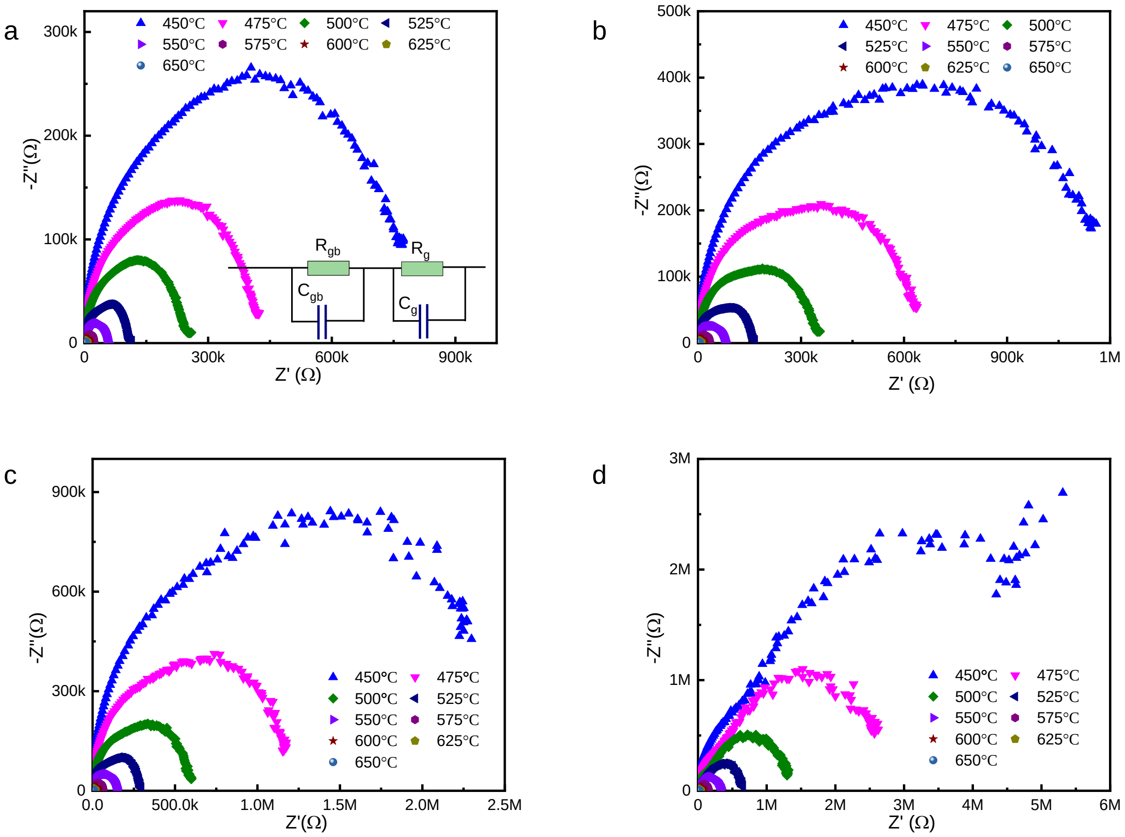

Figure S5. Impedance spectroscopy of $Ba_{0.4}Sr_{0.6-x}Ca_xNb_{2-x}Ta_xO_6$ (450 -650 °C). (a) $x = 0$, (b) $x = 0.15$, (c) $x = 0.30$, (d) $x = 0.45$ (the inset of (a)showing the equivalent circuit proposed for impedance data fitting).

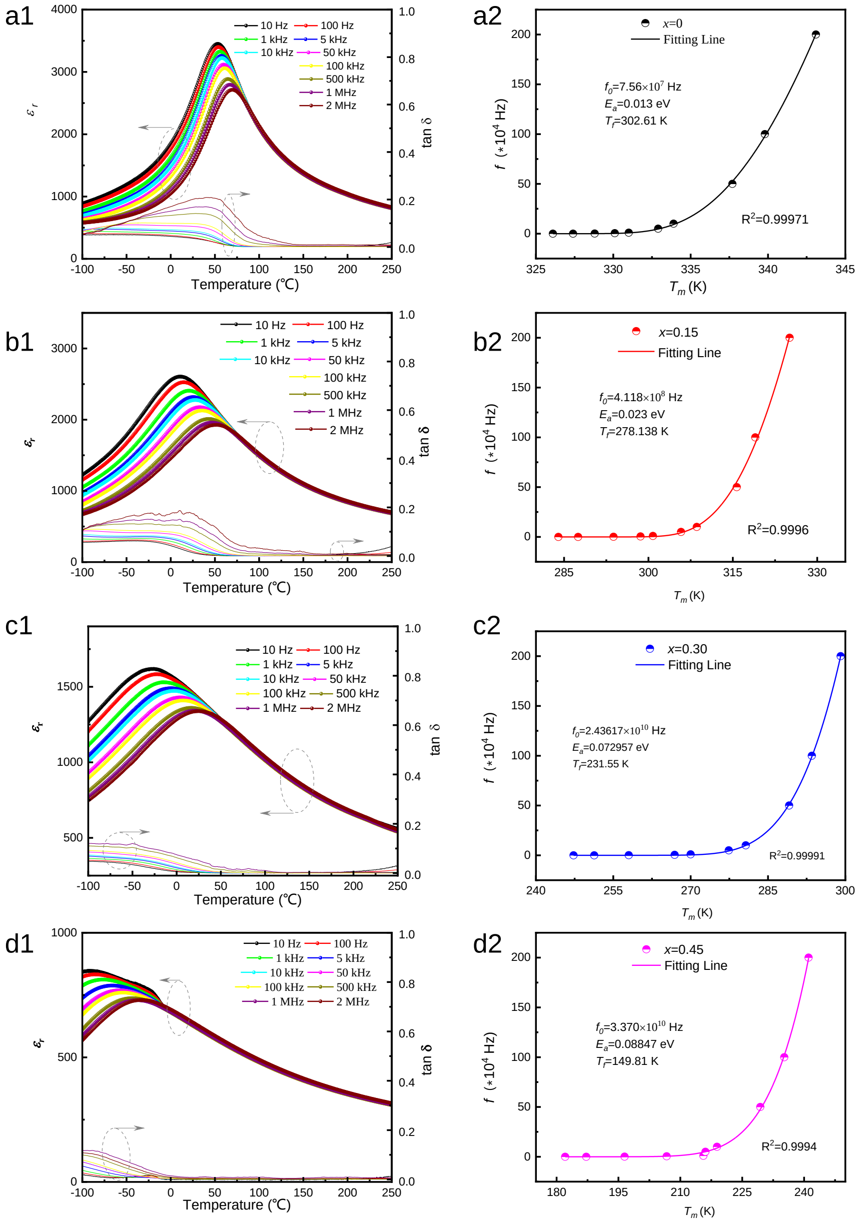

Figure S6. Dielectric properties of BSCNT*x* ceramics. (a)-(d) The temperature dependence of the dielectric constant (ε_r) and loss tangent (tanδ) for the BSCNT ceramics at various frequencies (10 Hz-2 MHz) and the frequency dependence of the temperature of the dielectric permittivity maximum (T_m) (the solid line denotes the fitting results to the Vogel-Fulcher relation).

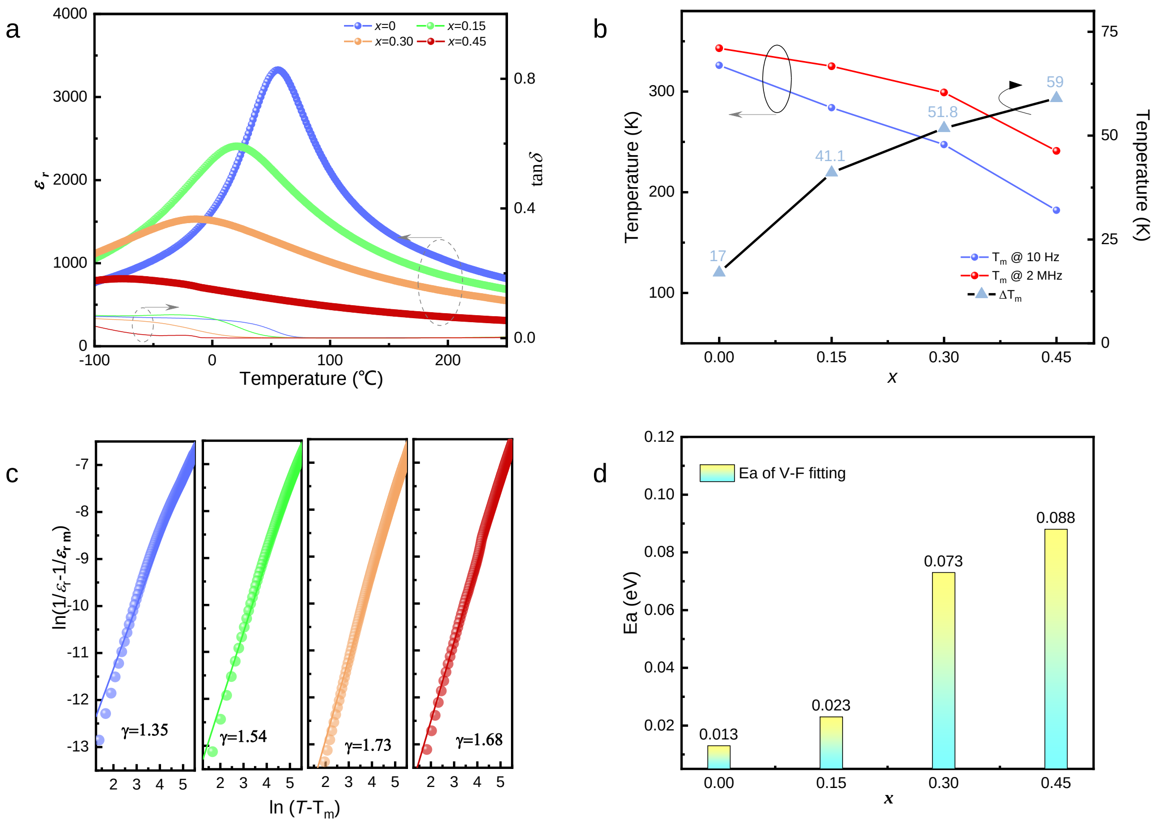

Figure S7. Relaxation properties of BSCNT*x* ceramics. (a)Temperature dependence of the dielectric constant and loss tangent (tanδ) of the BSCNT ceramics at 1 kHz. (b) T_m at 10 Hz and 2 MHz and the variation of T_m with a frequency. (c) The diffusion coefficient (γ) of BSCNT ceramics at 1kHz was obtained by using the modified Curie-Weiss formula. (d) The fitting result of E_a of the BSCNT ceramics by Vogel-Fulcher model.

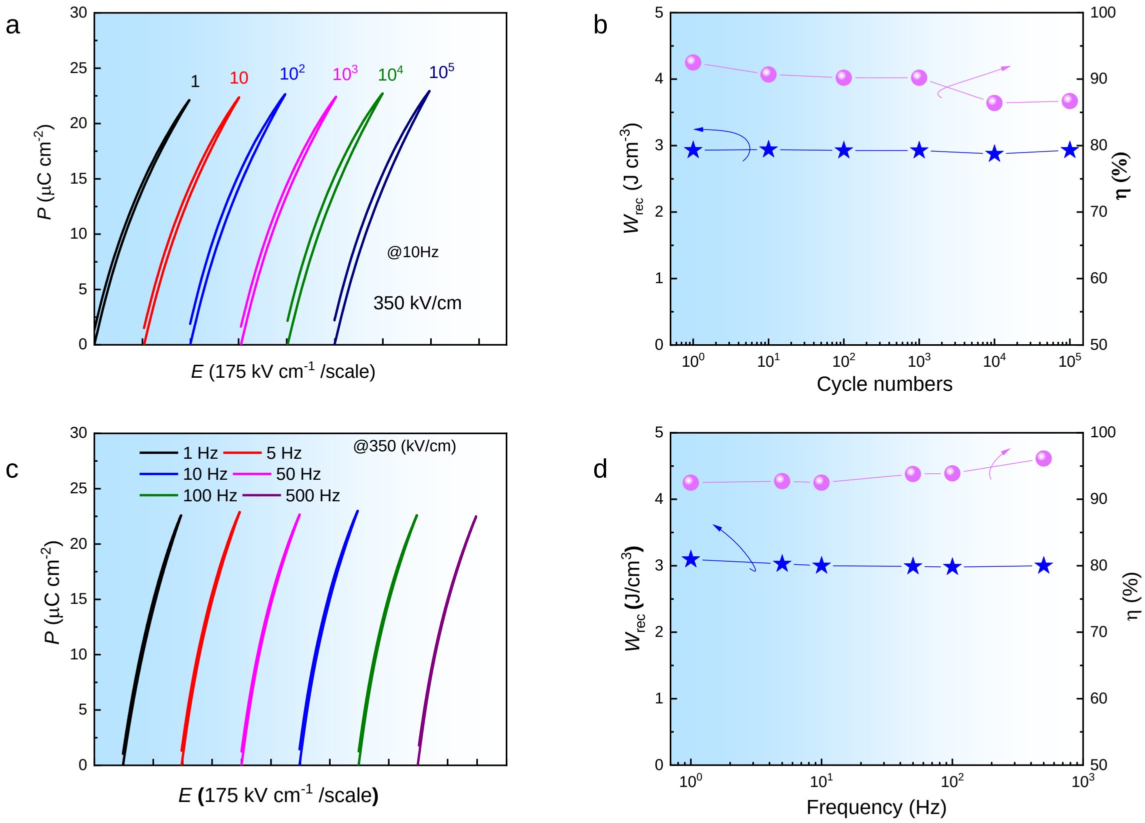

Figure S8. The stability of energy-storage performance of BSCNT0.30 samples. (a) *P-E* loops at 350 kV cm^{-1} with different cycles, (b) W_{rec} and η after different charge-discharge cycling numbers under 350 kV/cm, (c) *P-E* loops at 350 kV cm^{-1} with different frequencies. (d) W_{rec} and η with different frequencies.

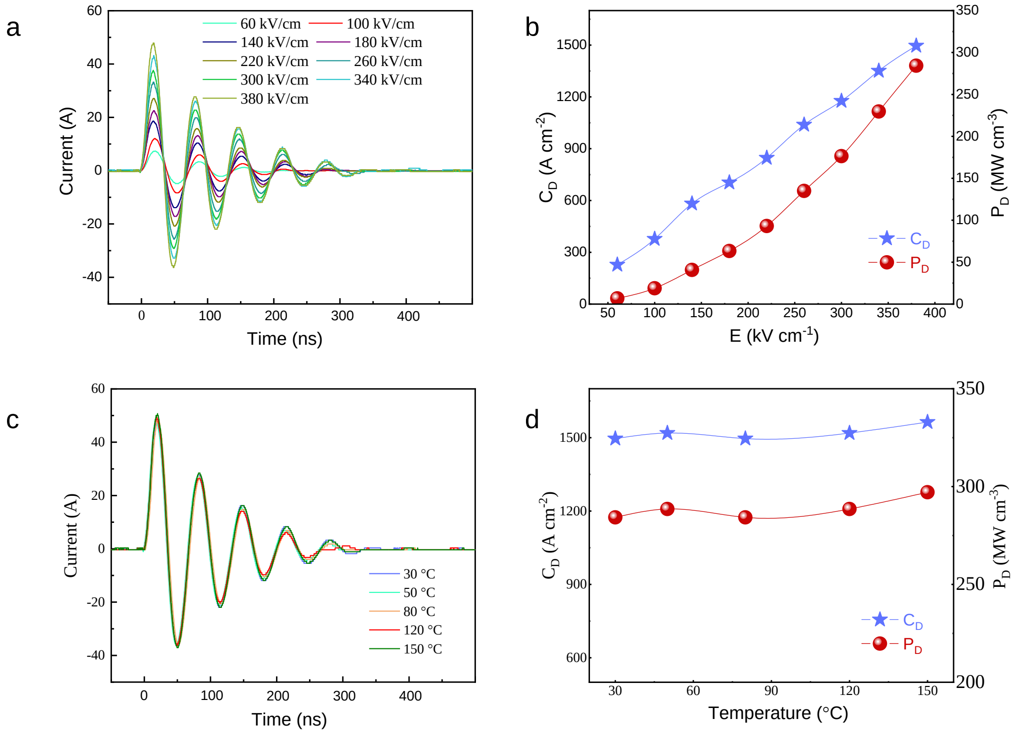

Figure S9. Underdamped charge-discharge performance of BSCNT0.30. (a) Underdamped charge-discharge curves of BSCNT0.30 ceramics at various electric fields. (b) C_D and P_D of BSCNT0.30 ceramics at various electric fields. (c) underdamped charge-discharge curves (@380 kV/cm) of BSCNT0.30 ceramics at various temperature. (d) C_D and P_D (@380 kV/cm) of BSCNT0.30 at various temperature.

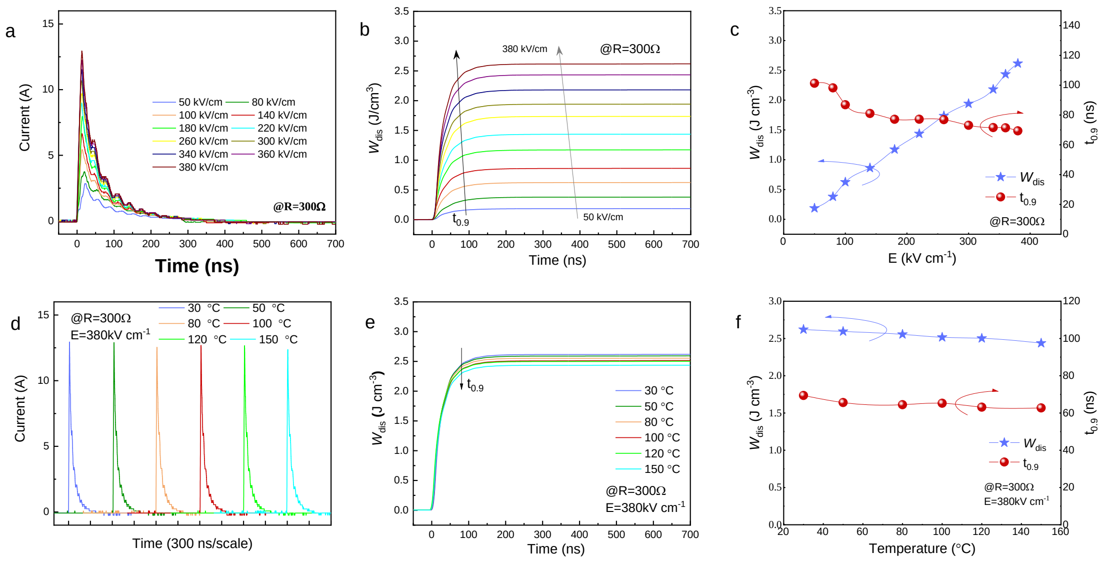

Figure S10. Overdamped charge-discharge performance of BSCNT0.30. (a) The overdamped charge-discharge as a function of time at various electric fields. (b) W_{dis} as a function of time at various electric fields. (c) The variation of W_{dis} and $t_{0.9}$ with electric field. (d) The overdamped charge-discharge as a function of time at various temperature. (e) W_{dis} as a function of time at various temperature. (f) The variation of W_{dis} and $t_{0.9}$ with temperature.

References

1. Zhi Y, Chen A, Vilarinho PM, Mantas PQ, Baptista JL. Dielectric relaxation behaviour of Bi:$SrTiO_3$: I. The low temperature permittivity peak. *J Eur Ceram Soc* **18**, 1613-1619 (1998).

2. Nayak S, *et al.* Effect of A-site substitutions on energy storage properties of $BaTiO_3$-$BiScO_3$ weakly coupled relaxor ferroelectrics. *J Am Ceram Soc* **102**, 5919-5933 (2019).

3. Qi H, Xie A, Tian A, Zuo R. Superior Energy-Storage Capacitors with Simultaneously Giant Energy Density and Efficiency Using Nanodomain Engineered $BiFeO_3$ -$BaTiO_3$-$NaNbO_3$ Lead-Free Bulk Ferroelectrics. *Adv Energy Mater* **10**, 1903338 (2020).

4. Irvine JTS, Sinclair DC, West AR. Electroceramics: Characterization by Impedance Spectroscopy. *Adv Mater* **2**, 132-138 (1990).

5. Kubelka P. Ein beitrag zur optik der farbanstriche. *Z tech Phys* **12**, 593-601 (1931).
